# Bilharziosis of the Appendix

**Published:** 1911-01-21

**Authors:** 


### Tropical Medicine.

### BILHARZIOSIS OF THE APPENDIX.

Turner, in the *Transvaal Medical Journal* for July 1910, shows that in certain native races inhabiting the East Coast of Africa, between 14° and 26° south, the appendix vermiformis is as frequently attacked by bilharzia as the urinary bladder. Out of a total of twenty-seven appendices examined no fewer than twenty were found on microscopical examination to be thus infected. In most of these the lesions were quite evident to the naked eye; on five occasions, for example, the mucous membrane was sanded; in another there was a small abscess cavity the size of a pea; in another the mucous membrane showed black powder grains; while in yet another regular warts were present. The coats of the infected appendices were as a rule thickened. The species of ova varied; in 85 per cent. of the cases terminal spined ones were present alone; in 5 per cent. lateral spined ones, with mixed infections in 10 per cent. The pathological changes produced in the appendix resembled macroscopically and microscopically the lesions found in the bladder much more closely than those found in the rest of the alimentary canal. Clinical manifestations were conspicuous by their absence; none of the cases, as far as could be determined, exhibited any of the usual signs of appendicitis.

